# Remote Sensing Image Classification Based on Canny Operator Enhanced Edge Features

**DOI:** 10.3390/s24123912

**Published:** 2024-06-17

**Authors:** Mo Zhou, Yue Zhou, Dawei Yang, Kai Song

**Affiliations:** College of Information Science and Engineering, Shenyang Ligong University, Shenyang 110159, China; zhoumo@sylu.edu.cn (M.Z.);

**Keywords:** remote sensing, scene classification, feature extraction, multi-level feature, feature fusion

## Abstract

Remote sensing image classification plays a crucial role in the field of remote sensing interpretation. With the exponential growth of multi-source remote sensing data, accurately extracting target features and comprehending target attributes from complex images significantly impacts classification accuracy. To address these challenges, we propose a Canny edge-enhanced multi-level attention feature fusion network (CAF) for remote sensing image classification. The original image is specifically inputted into a convolutional network for the extraction of global features, while increasing the depth of the convolutional layer facilitates feature extraction at various levels. Additionally, to emphasize detailed target features, we employ the Canny operator for edge information extraction and utilize a convolution layer to capture deep edge features. Finally, by leveraging the Attentional Feature Fusion (AFF) network, we fuse global and detailed features to obtain more discriminative representations for scene classification tasks. The performance of our proposed method (CAF) is evaluated through experiments conducted across three openly accessible datasets for classifying scenes in remote sensing images: NWPU-RESISC45, UCM, and MSTAR. The experimental findings indicate that our approach based on incorporating edge detail information outperforms methods relying solely on global feature-based classifications.

## 1. Introduction

Given the rapid advancements in aerospace technology, remote sensing has emerged as an indispensable tool for Earth observation missions. Based on remote sensing images, more research has been promoted for civil and military applications, such as urban planning, environmental monitoring, disaster assessment, etc. Analyzing these images plays an important social and economic role.

Researchers in the field of remote sensing have shown considerable interest in scene classification, which plays a crucial role in understanding images captured from a distance. The role it plays is of paramount importance in numerous Earth observation applications, and its extensive utilization can be observed in domains such as national defense and security, land use, urban planning, and geographic image retrieval, among others [1,2].

In recent years, deep learning has emerged as the prevailing trend in the domain of big data analysis and has achieved remarkable advancements across various computer vision tasks. The classification of remote sensing images showcases remarkable results with the utilization of the convolutional neural network (CNN), which autonomously captures and extracts advanced abstract features from raw data [3,4,5].

**Figure 1:**
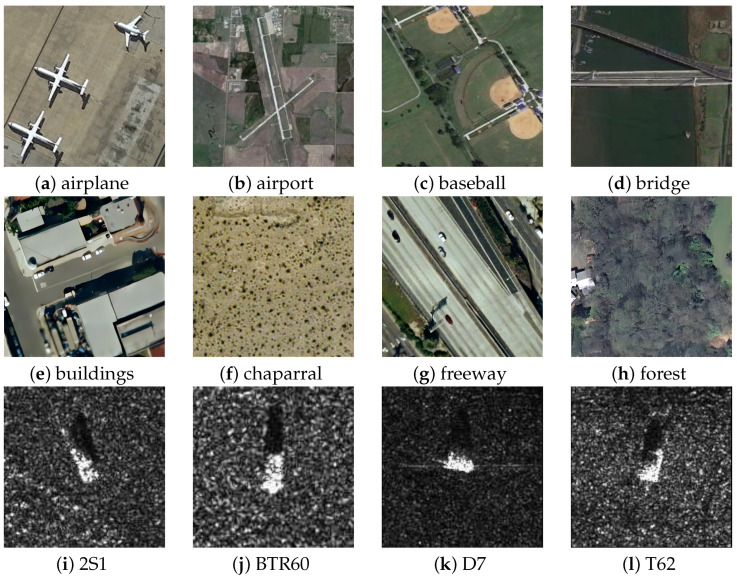
Example of images from different datasets. NWPU-RESISC45 (**a**–**d**); UCM (**e**–**h**); MSTAR (**i**–**l**).

The classification tasks in remote sensing imagery face new problems and challenges due to the ever-increasing scale of remote sensing image data. The image examples of commonly used remote sensing datasets are illustrated in Figure 1. Primarily, the majority of CNN approaches employ features extracted from the highest layer for classifying remote sensing images. The deep-level advanced image features, while providing abundant meaning and content information, typically focus solely on the local details or overall characteristics of an image, disregarding the interrelationships between different parts and the overall complex structure. Consequently, when confronted with images containing a substantial amount of information and intricate structures, the model’s recognition capability and classification accuracy may become constrained [6,7,8]. Secondly, with the diversity of remote sensing image data in different times, seasons, and regions, as well as changes in the perspective and scale of aerial images and satellite images, the appearance features and scales of objects such as shape, texture, and color change, which cause trouble for scene classification tasks.

In recent years, to address the issue of limited features for image classification, researchers have proposed multi-scale feature fusion and multi-modal information fusion techniques as means to expand and enrich the input features for image classification. Hong et al. [9] introduced a multimodal dataset for the classification of remote sensing data. Wu et al. [10] employed a cross-modal reconstruction strategy along with a sophisticated module called CCR-Net to achieve a more concise fusion representation of diverse remote sensing data sources. Bai et al. [11] presented a network for efficient multi-scale feature fusion, capturing features across various frequencies and scales for scene classification purposes, which proved advantageous by incorporating both high- and low-frequency characteristics to enhance classification accuracy. Yang et al. [12] proposed an enhanced multi-scale feature fusion network that effectively captures features through a parallel multi-path network. Ai et al. [6] proposed a novel approach that utilizes convolution kernels of varying dimensions to extract depth characteristics from SAR targets; the experimental results provide compelling evidence that the incorporation of multi-scale features enhances the robustness of SAR target recognition in the presence of speckle noise interference. Li et al. [13] devised a two-channel CNN architecture integrating attention map-derived features with RGB stream-based ones to extract fusion features, thereby demonstrating improved discriminative power. The aforementioned studies demonstrate that the integration of multiple information sources can effectively address the issue of limited input features in remote sensing image classification, enabling a more comprehensive and accurate representation of remote sensing image characteristics. Consequently, this approach enhances the performance and precision of remote sensing image classification, offering a viable resolution for examination and execution in the relevant field. However, the problem of underutilizing shallow features remains unresolved.

The classification performance of remote sensing image processing can be improved by extracting relevant prior information from large convolutional neural network models and imposing constraints on the network, thereby mitigating the adverse effects of appearance characteristics and scale changes of remote sensing objects on image processing results [13,14]. The efficacy of incorporating edge information for capturing image details has been validated in the context of remote sensing image segmentation tasks [15,16]. Wang et al. [17] proposed an edge enhancement channel attention mechanism, which selectively identifies effective channels after enhancing spatial edge features. This mechanism assists in the identification of blurred or irregular mining areas (MLC). Additionally, Hao et al. [18] and Zhang et al. [19] validated the effectiveness of this approach in remote sensing classification tasks.

Prior studies employ multi-level or multi-scale networks to extract more discriminative image features, utilizing weighted addition or concatenation for fusing features from different levels or scales to enhance image detail information and obtain more discriminative features, which are then fed into the classifier for image scene classification. However, this approach lacks contextual understanding and fails to fully consider the correlation between features, resulting in suboptimal performance for scene classification [20]. Moreover, the disregard for image content in edge information leads to subpar accuracy, and the inclusion of manual feature extraction in some studies limits their applicability as they lack end-to-end structures.

**Figure 2:**
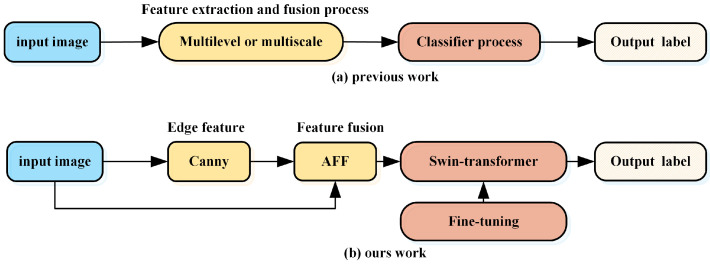
Illustration of the distinction between (**a**) previous works and (**b**) our work.

In this study, we propose a Canny edge-enhanced multi-level attention feature fusion network (CAF) to augment the extraction of more comprehensive features. The Canny operator is employed in our approach to extract edge information from images, effectively capturing diverse characteristics by integrating global information with edge details. We employ the AFF module, which effectively incorporates contextual information without introducing excessive parameters [21]. The original image data and edge information undergo multiple convolutions to derive shallow, medium, and deep features, respectively, followed by feature fusion using CAF structure. After feature fusion through CAF structure, they are inputted into the Swin-Transformer for feature extraction and classification. The comparative diagram illustrating the structural differences is presented in Figure 2.

The primary contribution of this article can be concisely summarized as follows:

(1) The proposed methodology utilizes a multi-level feature fusion approach to extract diverse features from remote sensing images. The integration of features at various levels facilitates the capture of intricate details as well as comprehensive contextual information within the image. Simultaneously, it facilitates the gradual extraction of abstract features, spanning from low-level to high-level representations, thereby yielding more comprehensive and semantically meaningful feature representations that facilitate enhanced interpretation and comprehension of the image.
(2) At each level, the edge information is simultaneously fused to enhance the representation of detailed features and achieve results.
(3) The CAF method, which fuses image features and edge features at each level, achieves better classification performance than without this method.

## 2. Related Work and Motivation

### 2.1. Related Work

**Hand-crafted features:** In the initial stages of remote sensing image scene classification, conventional hand-crafted features such as form, texture, hue, and spectral range are commonly utilized for feature extraction. The techniques employed for extracting hand-crafted features include color histogram, texture feature, Global Feature Information (GIST) [22], Gray Level Co-Occurrence Matrix (GLCM) [23], and Scale-Invariant Feature Transform (SIFT) [24,25], among others [26]. Despite their commendable stability and capacity to convey overall shallow information, these traditional hand-crafted features heavily rely on manual design and fail to effectively extract high-resolution remote sensing image features. As a result, their widespread application in classification tasks is limited.

**Ways to enhance feature information:** To enhance feature information for classification, a multi-branch [27,28,29], multi-level [30,31], and multi-scale [17,32] structure can be employed to fuse diverse features. Shi et al. [29] utilized a bilinear feature extraction structure to merge the extracted feature information from two branches, resulting in improved classification accuracy. Cheng et al. [31] incorporated a feature pyramid network and a squeeze-and-excitation block to obtain multi-level feature maps, while Shi et al. [33] proposed two convolutional combination modules for deep image feature extraction. The corresponding weights of all extracted features are calculated to facilitate fusion through multiple branches. Wang et al. [17] leveraged multi-scale convolution kernels to extract multi-scale information and introduced an additional branch of shallow features for fusion with deep features. However, when fusing features, employing ‘addition’ or ‘concatenation’ would directly result in a mere superposition or concatenation of information from distinct features, disregarding the inter-feature correlation. This oversight may blur the significance among different features and lead to redundancy or loss of crucial information.

**Loss function:** The remote sensing image classification tasks typically encounter the following challenges: (a) Acquiring images is often difficult, resulting in a limited number of samples for training sets [34]. (b) Some categories have an insufficient number of samples, leading to class imbalance issues. (c) Image interpretation poses difficulties and is susceptible to label noise interference and errors [35]. (d) Images exhibit significant intra-class differences and subtle inter-class distinctions, making fine-grained classification challenging. The aforementioned issues have prompted the development of diverse loss functions aimed at tackling these challenges. For classification problems that require precise distinctions or involve subtle differences, such as in SAR applications, incorporating L2 normalization into the cosine loss function acts as a robust regularizer and can effectively reduce the impact of misclassified samples, including challenging instances and label inaccuracies. Consequently, employing cosine loss [36] yields superior classification accuracy compared to standard cross-entropy loss. Moreover, it proves more suitable for small sample datasets like remote sensing images, which are typically arduous to acquire. The purpose of multi-class cross-entropy loss is to ensure equitable learning across all classes. However, in the presence of class imbalance during training, the model may exhibit excessive confidence towards the majority class and incorrectly classify most samples as belonging to this dominant category. This phenomenon can result in overfitting and have a negative impact on generalization performance. To address this problem, focal loss has been proposed as a solution that focuses on challenging categories [37]. The contribution of straightforward examples is diminished and more intricate classification instances are emphasized by focal loss, enabling a focused approach towards difficult-to-classify examples. By manipulating the regularization factor, it is possible to reduce the impact of simple examples at different levels. The Orthogonal Projection Loss (OPL) [38] is designed to optimize feature discrimination by maximizing inter-class orthogonality and minimizing intra-class distance, enhancing the model’s robustness against label noise and other practical interferences. This approach is particularly suitable for small sample datasets of remote sensing images.

### 2.2. Motivation

Currently, the majority of CNN-based models employ the ultimate classification features derived from the final stage for tasks that involve classifying images at a coarse-grained level. However, in tasks involving classification of fine-grained images, discarding the front-end shallow features can lead to a deterioration in classification accuracy, particularly when dealing with low-resolution (SAR) images. Furthermore, although shallow features can be propagated from the network’s initial layers to deeper ones through multiple convolutional and pooling operations, they may become diluted and consequently weaken the spatial expression capability of the final features. Additionally, issues such as small sample size and imbalance in remote sensing images also affect the accuracy of classification tasks.

The Swin-Transformer has gained significant traction among researchers in the field of remote sensing tasks due to its exceptional performance and ability to seamlessly integrate global and local data using window attention [30,39,40,41]. This is why we have chosen it as the backbone network to address the aforementioned challenges. To extract edge information, we utilize the Canny operator, which offers advantages such as multi-scale detection, noise reduction, precise localization, and an effective edge connectivity strategy. Additionally, we employ a multi-tier feature integration technique to extract diverse levels of features from remote sensing images. This approach is advantageous for capturing contextual information, extracting deep and shallow features, and improving model interpretability. By fusing features at different levels, both detailed information and global context can be captured while progressively extracting abstract features from low-level to high-level representations that are more comprehensive and semantically meaningful. Regarding fusion methods, we choose the attention-based image feature fusion technique due to its effectiveness in extracting and integrating crucial feature information from various regions within the image. This enables the model to focus better on significant regions and features within the image, thereby enhancing performance and robustness. Furthermore, by assigning appropriate weights to multiple loss functions, leveraging their unique advantages significantly enhances classification accuracy.

## 3. Method

### 3.1. Overall Framework

**Figure 3:**
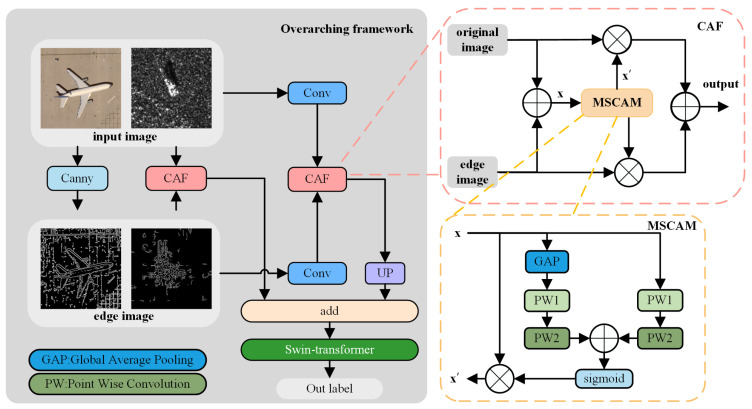
The overarching framework of CAF. The original image and edge image (with the same depth) are fused using the CAF module, ensuring that multi-layer features maintain their dimensions through upsampling. Subsequently, the resulting fusion features are fed as input into the Swin-transformer. The details of CAF and the multi-scale channel attention module (MSCAM) are also presented.

The overall structure of CAF is depicted in Figure 3. The fundamental elements of our network consist of a feature fusion module with multiple levels and a classifier for extracting features. Firstly, the edge image is obtained by applying the Canny edge detection operator on the original image, which is then combined with the original image using a dual branch structure. Convolution operations are performed iteratively on the dual branches to extract shallow and deep features separately (including global and detailed features). Subsequently, an AFF method proposed by Dai et al. [21] is employed to fuse corresponding level features from both branches. Afterwards, the fused information from multiple levels is superimposed to obtain enhanced edge information as well as discriminative shallow and deep characteristics. Finally, this output is fed into Swin-Transformer for further feature extraction and classification.

### 3.2. Multi-Level Feature Extraction

The process of multi-level feature extraction is illustrated in gray in Figure 3. The upper branch represents the multi-level extraction of figure features from the original image, while the lower branch represents the multi-level extraction of edge features. We perform convolutions once to extract features from both figures at different scales using a 3 × 3 convolution kernel, which helps avoid redundant network parameters and overfitting. Each convolution is followed by a maxpooling layer with a stride of 2, enabling size reduction of the feature map as the network deepens. To enhance abstract semantic features, we progressively increase channel width to 8, thereby minimizing the risk of feature loss.

The abovementioned approach integrates all extracted scales (features at different stages or depths) to facilitate feature fusion, thereby determining the ultimate classification outcome and ensuring enhanced representation capability.

### 3.3. Edge Information Enhancement

By utilizing the Canny operator to extract edge features from remote sensing objects, the global features of the image can be supplemented and further enhance classification performance. The extraction and complement process is illustrated in Figure 3.

Step 1: Inputting a remote sensing image figure, performing image sampling on each image, and resizing it to 224 × 224 size while maintaining the feature dimension unchanged.

Step 2: Conducting multi-layer feature extraction on both the original image and edge features obtained using the Canny operator at different scales (depths).

Step 3: Fusing the original image feature maps with corresponding scale (depth) edge feature maps (i.e., scales of 224 and 112). The specific fusion method will be described in detail in the subsequent section.

Step 4: Upsampling the scales (smaller than 224 × 224) to a size of 224 × 224 using an inversion convolution method. This step aims to utilize official weights trained on imagenet100k data for Swin-Transformer models, which can improve model performance by accelerating convergence and enhancing generalization abilities. Additionally, this approach reduces training time, making it suitable for small-scale datasets like remote sensing images that are not easily obtainable.

Step 5: Overlaying these four fused feature maps with different depths onto Swin-Transformer for conducting enhanced feature extraction and classification operations aiming at improving overall classification performance.

### 3.4. Feature Information Fusion Network

**Figure 4:**
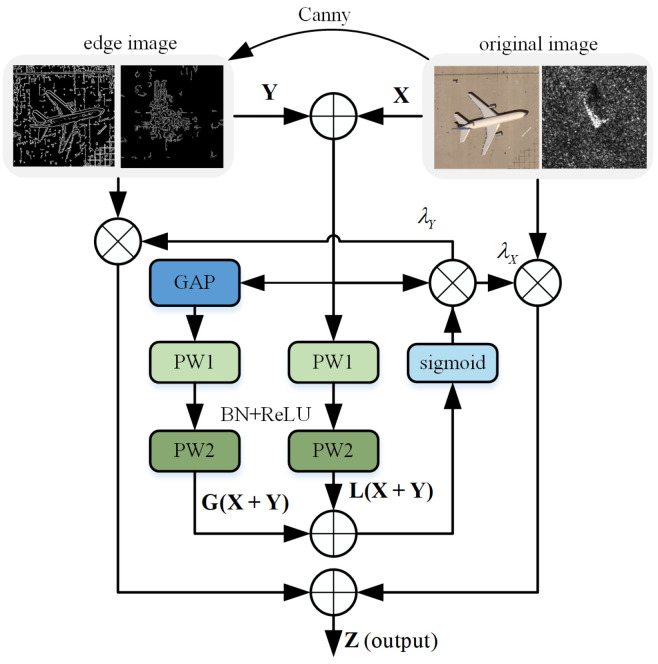
The proposed CAF. By employing the attention-based feature fusion approach, the weights $\lambda_{X}$ and $\lambda_{Y}$ are computed for integrating the original image and edge image. In comparison to the addition and concatenation methods, this technique enables enhanced focus on crucial regions and features within the image, thereby augmenting its performance and robustness.

We employ the Canny edge and AFF fusion method, referred to as CAF for short, as demonstrated in Figure 4 and Section 4 of this paper’s experiment, which successfully combines high-level and low-level characteristics from a single image to produce a depth fusion feature map by assigning suitable weights.

The feature **X** represents the Nth layer extracted from the original image, while **Y** represents the Nth layer extracted from the edge feature. Mathematically, **X** and **Y** can be represented as $X,Y\in R^{C\times H\times W}$. The original image feature **X** and edge feature **Y** are added together and divided into two branches, focusing on global and local information, respectively, which can be expressed as

(1) $$L\left(X+Y\right)=\mathrm{BN}({\mathrm{Conv}}_{2}(\mathrm{ReLU}\left(\mathrm{BN}\left({\mathrm{Conv}}_{1}\left(X+Y\right))\right)\right)$$

(2) $$G\left(X+Y\right)=\mathrm{BN}({\mathrm{Conv}}_{2}\left(\mathrm{ReLU}\left(\mathrm{BN}\left({\mathrm{Conv}}_{1}\left(g\left(X+Y\right)\right)\right)\right)\right)$$

After sigmoid, the feature weights of **X** and **Y** are generated, respectively, and the fusion feature map **Z** with weight information is generated—that is, image fusion feature based on the attention method is obtained.

Among them,

(3) $$\lambda_{X}=\mathrm{sig}\left(L\left(X+Y\right)⊕G\left(X+Y\right)\right)$$

(4) $$\lambda_{X}+\lambda_{Y}=1$$

The fused feature map $Z\in R^{C\times H\times W}$ can be represented as follows:

(5) $$Z=\lambda_{X}\left(X+Y\right)⊗X+\lambda_{Y}\left(X+Y\right)⊗Y$$

### 3.5. Loss Function

To adapt the loss function for remote sensing image applications, we combine multiple loss functions, including cosine loss, focal loss, and OPL loss, which are suitable for small sample sizes, class imbalances, and noisy labels commonly found in remote sensing images.

We use cosine loss to solve fine-grained classification problems and small-sample classification problems. In addition, this loss function is suitable for SAR images. It enhances the maximization of cosine similarity between the output of the neural network and one-hot vectors representing the true class. Consequently, employing cosine loss [36] yields superior classification accuracy compared to standard cross-entropy loss.

The calculation of cosine similarity for two vectors $a,b\in R_{d}$ in d-dimensional space is determined by the angle formed between them and defined as

(6) $$\sigma_{\cos}\left(a,b\right)=\cos\left(a∠b\right)=\frac{\langle a,b\rangle }{{∥a∥}_{2}\cdot {∥b∥}_{2}}$$

〈·,·〉 represents the dot product, while ${∥\cdot ∥}_{p}$ signifies the $L^{P}$ norm.

$\varphi_{\mathrm{onehot}}\left(y\right)$ refers to the establishment of a mapping between classes and the prediction space. The class embeddings φ are considered as fixed, and our goal is to acquire the parameters θ of a neural network $f_{\theta }$ by maximizing the cosine similarity between the features of images and their respective class embeddings.

(7) $$\varphi_{\mathrm{onehot}}\left(y\right)={\left[\underset{y-1\;\mathrm{times}}{\underset{︸}{0\cdots 0}}\;1\;\underset{n-y\;\mathrm{times}}{\underset{︸}{0\cdots 0}}\right]}^{T}$$

The cosine loss function, which is aimed to be minimized by the neural network, is defined for this purpose.

(8) $$L_{\cos}\left(x,y\right)=1-\sigma_{\cos}\left(f_{\theta }\left(x\right),\varphi \left(y\right)\right)$$

The neural network’s parameters θ are acquired through the maximization of cosine similarity between the features $f_{\theta }\left(x\right)$ and φ(y) extracted from images, and an instance x from a specific domain, where y represents the authentic classification of x chosen from a collection of categories.

To mitigate the detrimental impact of class imbalance on generalization performance during training, a potential solution known as focal loss has been proposed to specifically address overfitting issues associated with challenging categories [37].

The weight of the majority class is reduced in accordance with the cross-entropy loss, thereby directing the model’s focus towards learning the minority class. The cross-entropy loss can be expressed as follows:

(9) $$L_{\mathrm{entropy}}\left(\hat{y}\right)=-\alpha \log\left(\hat{y}\right)$$

The cross-entropy loss, μ, is augmented with a modulation factor ${\left(1-\hat{y}\right)}^{\mu }$, serving as an adaptable focusing parameter.

The Focal loss function allows for the weighting of losses based on the extent of prediction errors, exhibiting varying rates of loss weighting in response to different values of μ. In cases where errors are pronounced, specifically when the predicted label $\hat{y}\;$ tends towards 0, the Focal loss adjusts the weight assigned to these errors by increasing their significance. Consequently, the model allocates greater attention to these challenging samples. By diminishing the contribution of straightforward examples and emphasizing more intricate classification instances, Focal loss facilitates a focused approach towards difficult-to-classify examples.

The presence of label noise interference in remote sensing image interpretation poses a significant challenge. The Orthogonal Projection Loss (OPL) [38] is designed to enhance the model’s robustness against label noise and other practical interferences, making it particularly suitable for small sample datasets of remote sensing images.

(10) $$L_{\mathrm{OPL}}=\left(1-s\right)+\beta *|d|$$

The objective of OPL loss is to enforce the functionality of constrained clustering, ensuring that the features pertaining to different classes are orthogonal in the feature space while exhibiting similarity within the same class. This serves to enhance the discriminative capability of the model.

The total loss, referred to as COFE loss, is a weighted combination of these image classification losses and can be precisely defined as follows:

(11) $$L_{\mathrm{COFE}}=L_{\mathrm{entropy}}+\delta \cdot L_{\cos}+\eta \cdot L_{\mathrm{focal}}+\lambda \cdot L_{\mathrm{OPL}}$$

## 4. Experiments and Result

The performance of our CAF method in classifying remote sensing data is assessed by conducting experiments on three publicly accessible datasets in this section: the University of California Merced Land Use (UCM) Data Set [42], the Northwestern Polytechnical University Remote Sensing Image Scene Classification 45 (NWPU RESISC45) Data Set [2], and the Moving and Stationary Target Acquisition and Recognition (MSTAR) Data Set [43]. The main information of the three datasets is shown in Table 1.

### 4.1. Experimental Details

The input images were standardized to a resolution of 224 × 224 pixels as a preliminary step in the training process. To bolster the model’s efficacy and counteract the potential for overfitting, we implemented the RandomResizedCrop data augmentation strategy [44,45]. This technique has a proven track record for refining model performance and alleviating overfitting tendencies. We meticulously selected the following hyperparameters: a batch size of 64 and a learning rate of 0.0001. A wealth of empirical evidence has corroborated the enhanced performance delivered by these particular parameter settings. The epoch number is 100. For the optimization of weight parameters across all layers, we employed the Adam Weight Decay (AdamW) optimizer, continuing the optimization process until the model converged [46,47,48].

The PyTorch-based experiments detailed herein were conducted on a personal computing platform equipped with an Intel i7-12700 CPU, an NVIDIA GeForce RTX 3090 graphics card, and 24 GB of RAM.

The efficacy of our methodology was rigorously assessed by segregating the dataset into training and testing subsets, adhering to the conventional partitioning ratios. The dataset was allocated an 80% ratio for training purposes. Ablation studies were meticulously executed to substantiate the performance improvements conferred by the integration of the CAF fusion network and the novel loss function, specifically in relation to classification accuracy.

Regarding the selection of hyperparameters in the COFE loss function, all four types of losses are assigned equal weights of 1, and the hyperparameters for each type of loss function are determined based on the values provided in the original literature [36,37,38].

### 4.2. Performance Evaluation Metrics

The performance of the proposed CAF method for scene classification is quantitatively evaluated by employing several commonly used metrics as evaluation indices to assess experimental results. The metrics encompassed in this study comprise overall accuracy (OA), Kappa coefficient (Kappa), and the confusion matrix (CM), enabling both quantitative and qualitative comparisons of classification performance.

The Confusion Matrix serves as a reflection of the classification outcomes, providing a foundation for comprehending other image classification evaluation metrics. In cases where there are n sample classes, the confusion matrix C is represented by an n×n square matrix, with its expression depicted in Formula (12).

(12) $$CM=\left[\begin{array}{cccc} c_{11} & c_{12} & \cdots & c_{1n}\\ c_{21} & c_{22} & \cdots & c_{2n}\\ \cdots & \cdots & \cdots & \cdots \\ c_{n1} & c_{n2} & \cdots & c_{nn} \end{array}\right]$$

The value of $c_{ij}$ denotes the count of instances belonging to class *i* that were assigned to class *j*. The total number of class *i* ground feature samples can be denoted by $\sum_{i=1}^{n}c_{ij}$, and $\sum_{j=1}^{n}c_{ij}$ represents the number of samples classified as class *j* ground features.

Formula (13) demonstrates the calculation method for overall accuracy (OA), which is determined by dividing the sum of correctly classified samples $\sum_{i=1}^{n}c_{ii}$ by the total number of samples $\sum_{j=1i=1}^{n}\sum_{ij}^{n}c_{ij}$. The overall accuracy serves as an indicator of the classifier’s performance as a whole, yet it is significantly influenced by imbalanced sample distribution, particularly when one class dominates with a large number of samples.

(13) $$OA=\frac{\sum_{i=1}^{n}c_{ii}}{\sum_{j=1}^{n}\sum_{i=1}^{n}c_{ij}}$$

The Kappa coefficient plays a vital role in assessing the effectiveness of image classification by measuring the level of concordance between classification outcomes and actual values. It is calculated using Formula (14). The Kappa coefficient represents the extent to which the current classification method reduces errors compared to a completely random classifier, with values typically ranging from 0 to 1. The higher the Kappa coefficients, the stronger the consistency and enhanced model performance in classification.

(14) $$Kappa=\frac{N\sum_{i=1}^{n}c_{ii}-\sum_{i=1}^{n}\left(\sum_{j=1}^{n}c_{ij}\times \sum_{i=1}^{n}c_{ij}\right)}{N^{2}-\sum_{i=1}^{n}\left(\sum_{j=1}^{n}c_{ij}\times \sum_{i=1}^{n}c_{ij}\right)}$$

In addition, we compared alternative evaluation metrics for assessing the classification performance, namely, Precision, Recall, and F1 Score (F1). The calculation method for these four metrics is defined by Equations (15)–(17), respectively. In this context, *TP* represents True Positive, denoting accurately classified positive samples. The abbreviation *TN* stands for True Negative, which refers to accurately classified negative samples. *FP* represents False Positive, indicating the misclassification of positive samples that are actually negative. *FN* denotes False Negative, representing the misclassification of negative samples that are actually positive.

(15) $$Precison=\frac{TP}{TP+FP}$$

(16) $$Recall=\frac{TP}{TP+FN}$$

(17) $$F_{1}=\frac{Precison\times Recall}{0.5\times (Precision+Recall)}$$

### 4.3. Results

We conducted a series of experiments using the Swin-Transformer as the sole baseline for feature extraction and classification. These experiments included the following: (1) sole use of the Swin-Transformer with cross-entropy loss function; (2) utilization of the novel loss function in conjunction with the Swin-Transformer; (3) integration of CAF fusion with the Swin-Transformer along with cross-entropy loss function; (4) fusion of CAF with the Swin-Transformer while employing the novel loss function; (5) application of CAF twice alongside cross-entropy loss function in combination with the Swin-Transformer; and, finally, (6) dual utilization of CAF fusion along with application of the novel loss function on top of the Swin-Transformer. The findings from the experiments are displayed in Table 2, Table 3 and Table 4.

By analyzing Table 2, it can be observed that the influence of the loss function on the classification outcome is minimal when solely employing the Swin-Transformer as a classifier for original image classification. This phenomenon arises due to our proposed novel loss function primarily addressing challenges associated with small sample sizes and imbalanced datasets. Consequently, improvements in accuracy are evident on the UCM dataset characterized by a lower number of samples per class, while the accuracy rate remains almost unchanged on the other two datasets.

Furthermore, through the analysis of Table 3 and Table 4, it is apparent that incorporating edge information in the CAF method indeed enhances classification accuracy compared to direct utilization of original images for classification purposes. Moreover, mining depth information with each additional convolutional layer further improves accuracy levels, as evidenced by experiments conducted on visible light datasets. However, intriguingly, SAR dataset analysis reveals that mining only one layer of deep information yields higher accuracy than mining two layers; we attribute this discrepancy to unique image characteristics inherent in SAR data.

**Figure 5:**
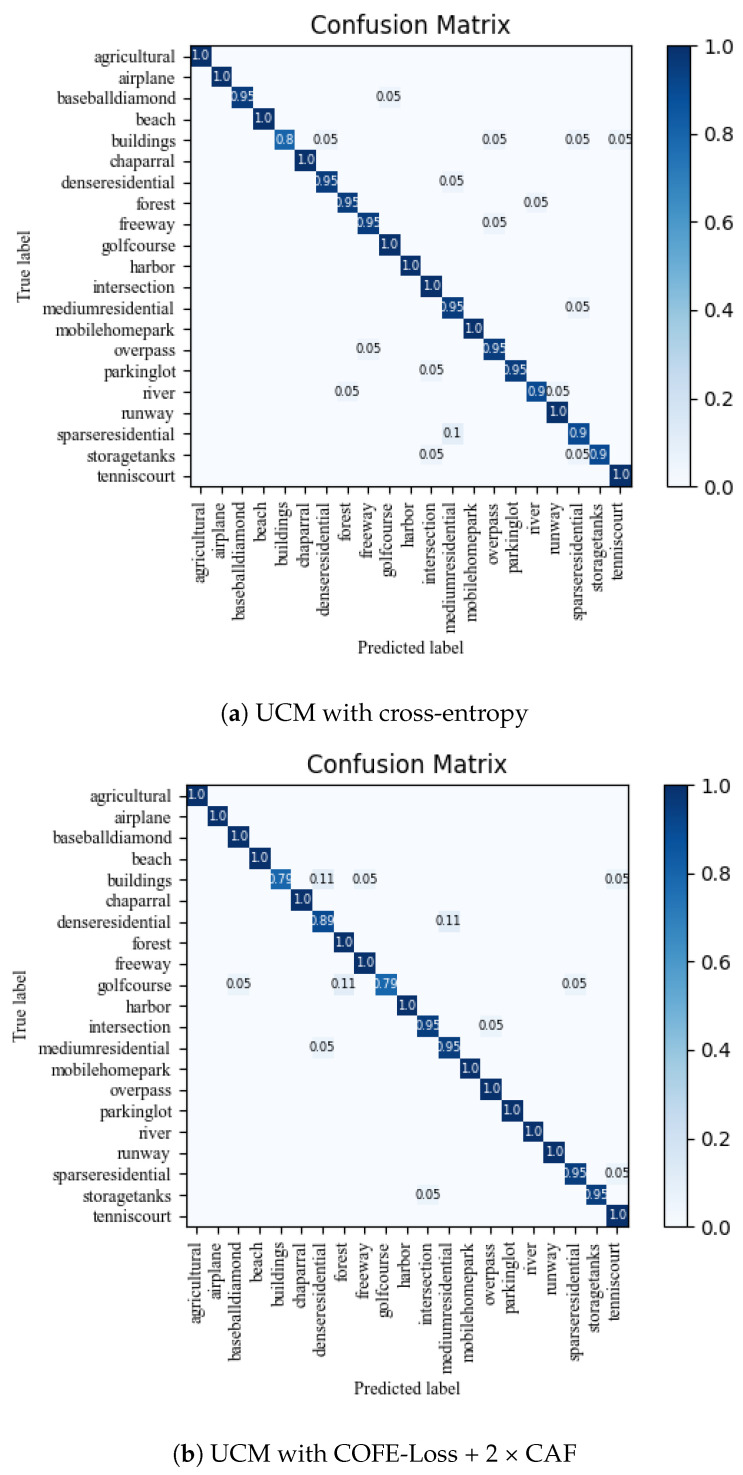
The confusion matrices of UCM were computed using two different methods, with a training ratio of 80%.

**Figure 6:**
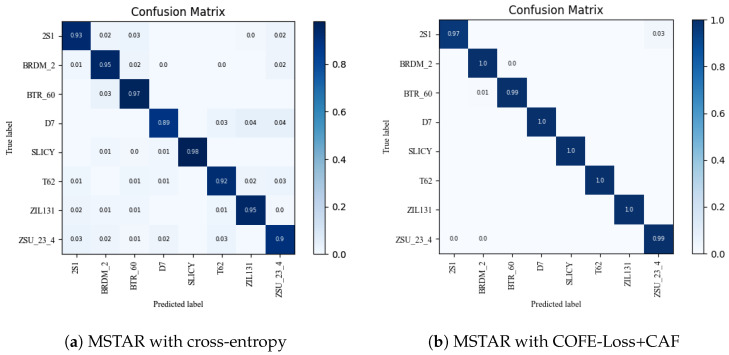
The confusion matrices of MSTAR were computed using two different methods, with a training ratio of 80%.

**Figure 7:**
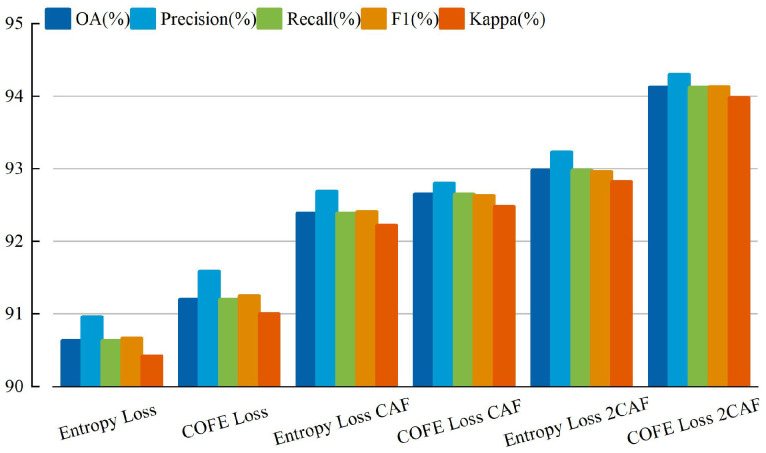
The assessment metrics of NWPU-RESISC45 employing diverse methodologies.

**Figure 8:**
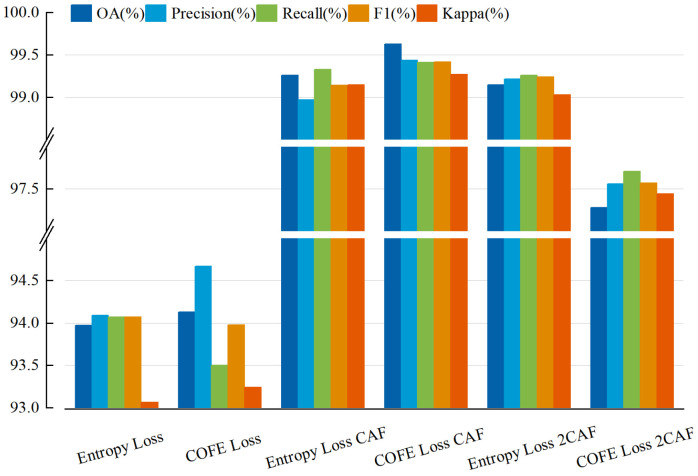
The assessment metrics of MSTAR employing diverse methodologies.

**Figure 9:**
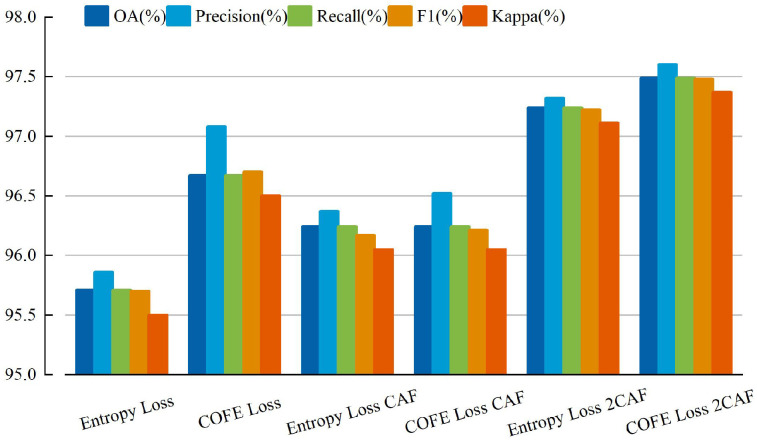
The assessment metrics of UCM employing diverse methodologies.

In addition, as shown in Figure 5 and Figure 6, we conducted an analysis of the enhancements for each category in the UCM and MSTAR datasets. Notably, Figure 5 and Figure 6 demonstrates a significant reduction in misclassified samples and scenes when employing our method, thereby substantiating the efficacy of our proposed approach. The evaluation metrics for the three datasets using different methods are presented in Figure 7, Figure 8 and Figure 9 and Table 5.

To acquire a holistic comprehension of the influence exerted by the CAF technique on tasks related to classification, we present feature heat maps for target samples generated by networks with and without the CAF module. This analysis aims to provide valuable insights into how this method influences classification performance and can contribute to future research in this field.

**Figure 10:**
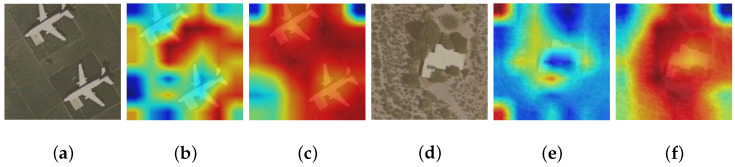
The sequence from (**a**–**f**) includes the UCM dataset’s initial image, the heat map created by the model without incorporating the CAF module (overlaid on top of the original image), and the heat map generated by integrating the CAF module into the model (also overlaid on top of the original image).

**Figure 11:**
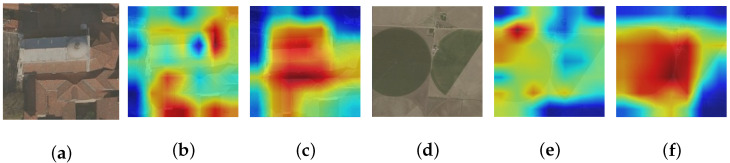
The sequence from (**a**–**f**) includes the NWPU-RESISC45 dataset’s initial image, the heat map created by the model without incorporating the CAF module (overlaid on top of the original image), and the heat map generated by integrating the CAF module into the model (also overlaid on top of the original image).

**Figure 12:**
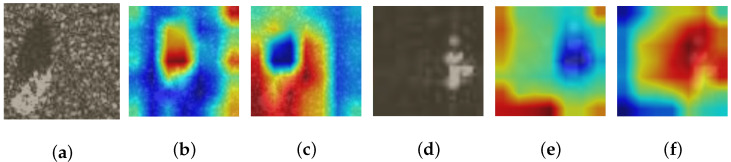
The sequence from (**a**–**f**) includes the MSTAR dataset’s initial image, the heat map created by the model without incorporating the CAF module (overlaid on top of the original image), and the heat map generated by integrating the CAF module into the model (also overlaid on top of the original image).

The heat maps of UCM, NWPU-RESISC45, and MSTAR datasets are displayed in Figure 10, Figure 11 and Figure 12. Two samples from each dataset are provided as examples, with each sample consisting of three images. The arrangement from left to right consists of the original image, the heat map generated by the model excluding the CAF module (superimposed on the original image), and the heat map produced by incorporating the CAF module into the model (also superimposed on the original image). Darker colors (blue) indicate lower activation values and smaller contributions while brighter colors (red) represent higher activation values and greater contribution to classification. Thus, significant regions that contribute to classifying images based on our model’s decision-making process can be observed. As depicted in Figure 10, Figure 11 and Figure 12, models that do not incorporate a CAF module have two main issues arise: Firstly, activation regions mainly concentrate on background areas rather than accurately identifying foreground objects, such as an airplane in Figure 10a, a house in Figure 10d, a church in Figure 11a, or circular farmland in Figure 11d. This occurs because integrating a CAF module encourages network exploration of potential domain invariant features through edge extraction and feature fusion resulting in more discriminative features. Secondly, for images within the MSTAR dataset that exhibit prominent shadows, like Figure 12a,d, models lacking a CAF module tend to prioritize shadows over target identification; however, employing a CAF module helps alleviate this issue.

The classification accuracy metric is employed as an objective indicator to assess the performance of our proposed model. To thoroughly analyze the strengths and weaknesses of our model, we perform a comparative evaluation of the proposed method against several state-of-the-art algorithms on the three datasets. The results are given in Table 6. The superiority of our method is evident, as it surpasses the majority of existing methods.

To objectively and comprehensively demonstrate the superiority of the proposed CAF, an ablation experiment was conducted, encompassing four conditions: ➀ employing solely the Swin-transformer, ➁ utilizing a Swin-transformer-based approach with enhanced edge features through addition, ➂ integrating CAF with the Swin-transformer, and ➃ combining two instances of CAF with the Swin-transformer. To ensure the fairness of the experiment, we used the cross-entropy loss function. The experimental findings unequivocally validated that incorporating edge information via the CAF method surpassed the mere addition of edge information. The results are given in Table 7.

## 5. Discussion

Experimental results demonstrate significant improvements in classification accuracy and discriminability with our approach. Specifically, compared to the baseline on different datasets, our method achieves respective improvements of 5.66%, 3.49%, and 1.78%, validating its effectiveness.

By incorporating image edge information, attention is visibly focused on objects with clear edges in the generated heat maps. The proposed enhancement significantly enhances recognition performance, particularly in scenarios characterized by a simple background or distinct objects to be recognized. This approach effectively improves overall recognition performance, as demonstrated on the MSTAR dataset. Moreover, when applied to the NWPU-RESISC45 dataset with a large number of samples, the proposed model also acquires more generalized features and enhances classification accuracy. However, challenges arise in complex scenes (e.g., buildings in UCM) or objects with simple shapes and uniform backgrounds (e.g., golf course in UCM), as these situations hinder effective learning of edge features and limit performance improvement.

Simultaneously, based on the outcomes of comparative experiments, we observed that the efficacy of mining two layers of feature information may not necessarily surpass that of mining a single layer, contingent upon the dataset’s characteristics. Consequently, in practical applications, it becomes imperative to make choices based on contextual factors.

Furthermore, the loss function proposed in this study exhibits distinct effects on enhancing classification accuracy owing to the dataset’s inherent characteristics. Ablation experiments reveal a notable enhancement in classification accuracy, particularly for datasets with limited samples and imbalanced distributions.

## 6. Conclusions

The article proposes a novel approach for classifying remote sensing scenes by extracting comprehensive features from remote sensing images through the integration of multi-layer global features and multi-layer edge features. To address the limitations of existing methods, such as inadequate utilization of depth information, neglecting edge information and image content, as well as insufficient consideration for contextual information and feature correlation during the fusion process, we introduce an edge information enhancement module, a multi-level feature extraction module, and a feature information fusion module. Experimental results demonstrate significant improvements in classification accuracy and discriminability with our approach. Furthermore, without the CAF architecture network design, the total number of parameters reached 27.53 M; subsequently, adding one or two CAFs increased the parameters by 21.318 M and 21.328 M, respectively. Further efforts are needed to optimize the network architecture for lightweight performance improvement.

## Figures and Tables

**Table 1:** Dataset description.

Datasets	Remote Sensing Imaging Types	Number of Classes	Number of per Class	Numbers of Instances	Image Size	Pixel Resolution	Year
NWPU-RESISC45	Very High-Resolution	45	700	31,500	256 × 256	0.2–30 m	2017
UCM	Very High-Resolution	21	100	2100	256 × 256	0.3 m	2010
MSTAR	Synthetic Aperture Radar	8	428–573	5172	368 × 368	0.3 m	1996

**Table 2:** Comparison of classification accuracy using the cross-entropy loss function and a novel loss function across three datasets.

Method	NWPURESISC 45	UCM	MSTAR
cross-entropy	90.63%	95.71%	93.97%
COFE-Loss	**91.20%**	**96.67%**	**94.13%**

**Table 3:** Comparison of classification accuracy using the cross-entropy loss function across three datasets.

Method	NWPURESISC 45	UCM	MSTAR
cross-entropy	90.63%	95.71%	93.97%
cross-entropy + CAF	92.39%	96.24%	**99.26%**
cross-entropy + 2 × CAF	**92.98%**	**97.24%**	99.15%

**Table 4:** Comparison of classification accuracy using a novel loss function across three datasets.

Method	NWPURESISC 45	UCM	MSTAR
COFE-Loss	91.20%	96.67 %	94.13%
COFE-Loss + CAF	92.65%	96.24%	**99.63%**
COFE-Loss + 2 × CAF	**94.12%**	**97.49%**	97.28%

**Table 5:** Numeric results of all metrics for all datasets.

Dataset	Method	Metric				
		Accuracy%	Precision%	Recall%	F1%	Kappa%
NWPU	cross-entropy	90.63	90.96	90.63	90.67	90.42
	COFE-Loss	91.20	91.59	91.20	91.25	91.00
	cross-entropy + CAF	92.39	92.69	92.39	92.41	92.22
	COFE-Loss + CAF	92.65	92.80	92.65	92.63	92.48
	cross-entropy + 2 × CAF	92.98	93.23	92.98	92.96	92.82
	**COFE-Loss + 2 × CAF**	**94.12**	**94.30**	**94.12**	**94.13**	**93.98**
MSTAR	cross-entropy	93.97	94.09	94.07	94.07	93.06
	COFE-Loss	94.13	94.67	93.50	93.98	93.24
	cross-entropy + CAF	99.26	98.97	99.33	99.14	99.15
	**COFE-Loss + CAF**	**99.63**	**99.44**	**99.41**	**99.42**	**99.27**
	cross-entropy + 2 × CAF	99.15	99.22	99.26	99.24	99.03
	COFE-Loss + 2 × CAF	97.28	97.56	97.71	97.57	97.44
UCM	cross-entropy	95.71	95.86	95.71	95.70	95.50
	COFE-Loss	96.67	97.08	96.67	96.70	96.50
	cross-entropy + CAF	96.24	96.37	96.24	96.17	96.05
	COFE-Loss + CAF	96.24	96.52	96.24	96.21	96.05
	cross-entropy + 2 × CAF	97.24	97.32	97.24	97.22	97.11
	**COFE-Loss + 2 × CAF**	**97.49**	**97.60**	**97.49**	**97.48**	**97.37**

**Table 6:** Classification accuracy of various targets with different methods.

Method	NWPURESISC 45	UCM	MSTAR
Res-Net50 [49]	94.96%	97.35%	53.80%
densenet121 [50]	94.90%	96.82%	64.56%
VGG11 [51]	93.56%	79.36%	58.92%
EMTCAL [7]	92.31%	96.25%	99.41%
ours method	**95.04%**	**97.49%**	**99.63%**

**Table 7:** Result of the ablation experiment.

Method	NWPURESISC 45	UCM	MSTAR
Swin-transformer	90.63%	95.71%	93.97%
add + Swin-transformer	90.28%	96.24%	98.15%
CAF + Swin-transformer	92.39%	96.24%	**99.26%**
2 × CAF + Swin-transformer	**92.98%**	**97.24%**	99.15%

## Data Availability

Data is contained within the article.
